# Exploring patient and clinician opinions, perspectives and acceptance of the use of artificial intelligence in the histological diagnosis of prostate cancer

**DOI:** 10.1002/bco2.70108

**Published:** 2025-11-09

**Authors:** Lisa Browning, Abhisek Ghosh, Monica Dolton, Kate Hutton, Jacqueline Birks, Richard Scheffer, Ewart Stanislaus, James Crofts, Richard Colling, Richard Bryant, Clare Verrill

**Affiliations:** ^1^ Department of Cellular Pathology Oxford University Hospitals NHS Foundation Trust Oxford UK; ^2^ Nuffield Department of Surgical Sciences University of Oxford Oxford UK; ^3^ NIHR Oxford Biomedical Research Centre Oxford University Hospitals NHS Foundation Trust Oxford UK

**Keywords:** artificial intelligence, clinician, diagnosis, histopathology, patient, prostate cancer, trust, urology

## Abstract

**Objectives:**

This study aims to explore the opinions and attitudes of patients and clinicians regarding the use of artificial intelligence (AI) in the diagnosis of prostate biopsies, with a focus on acceptance and trust in the use of AI, and factors that may impact this.

**Subjects and methods:**

Surveys were sent to patient members of UK‐based prostate cancer support groups and to a group of clinicians managing patients with prostate cancer (or suspected prostate cancer).

**Results:**

Of 130 patient respondents, 94% expressed acceptance of AI assistance in the diagnosis of prostate biopsies when a pathologist retains responsibility for the final diagnosis, but regard it as the responsibility of the pathologist to decide whether AI is used in this setting. Similar responses were noted among the nine clinician respondents. Regarding factors with potential impact on acceptance of AI, an understanding of how the AI was tested and its performance in comparison with a pathologist was considered to be more important than how the technology was developed, and half (51%) of patients suggested that access to additional information might impact their acceptance of AI.

**Conclusion:**

Understanding the perspectives of stakeholders is key to the successful clinical implementation of AI in the histological diagnosis of prostate biopsies. Our study shows a high level of acceptance of AI for the diagnosis of prostate biopsies among patients if a pathologist retains oversight of the diagnosis and the decision as to when AI is used. Furthermore, it suggests similar levels of acceptance among clinicians. Our study provides insight into areas for educational focus to enhance understanding of AI in this setting.

## 1 INTRODUCTION

The potential utility of artificial intelligence (AI) within urological practice is diverse, with roles anticipated in diagnosis and prognostication, treatment planning and delivery, training, enhanced performance of clinical trials, and efficiency gains through automation^1^, ^2^, ^3^, ^4^, offering real potential for the transformation of clinical practice. Yet there is much still to learn about the real‐world application of AI, particularly regarding its acceptance among clinicians and within the patient and public sector.

Most AI applications are still within the research or validation phase; however, AI applications in prostate cancer are leading the way in the field and we are beginning to see a small number implemented within a clinical workflow. These real‐world applications are currently limited largely to roles in histological diagnosis, and AI‐assistance tools for the diagnosis of prostate biopsies (cancer detection and grading) have been among the first in the world to receive accreditation for use in clinical practice with several systems in clinical use^5^, ^6^, ^7^, which show promise in potential improvements in pathologist efficiency and diagnostic accuracy^5^, ^6^, ^7^, ^8^, ^9^, ^10^. This looks to be particularly significant given that the current estimated 15 million prostate biopsy samples worldwide per annum^7^ is predicted to increase, at a time when histopathology is faced with continued challenges in the workforce^11^; AI is forecasted to have a significant impact on histopathology within the coming decade^12^.

The development of AI as a predictive tool for prostate cancer is another significant advance,^3^, ^7^ with AI models for the prediction of prostate cancer outcome and potential treatment responsiveness now clinically available^13^. While it is very early days for the real‐world use of such AI, the most recent National Comprehensive Cancer Network (NCCN) guidelines (version 4.2024)^14^ recommend the use of advanced risk stratification tools which include AI digital pathology (ArteraAI) ‘when they have the potential ability to change disease management’, in view of the demonstrated improved prognostication over that offered by standard tools^13^ through the utilisation of digitised patient biopsy images integrated with clinical data.

Beyond histological diagnosis, AI in the imaging detection of prostate cancer is also on the cusp of becoming a clinical reality^15^. A recent international study comparing radiologists versus AI for the detection of clinically significant prostate cancer on MRI has shown promise in the performance of AI^16^, with prospective studies now needed to take this forward in the clinical setting^17^. Looking beyond this, AI in combination with traditional prognostic pathological parameters, imaging, genomics, and clinical data has the potential power to offer even further improvement in personalised care, aiding treatment decision‐making, risk prediction and prediction of treatment responsiveness^3^, ^7^. Such AI models may begin to address the deficiencies recognised in current risk stratification models^18^ with consequent benefit to patient outcome.

With these advances in AI in mind, urologists and other members of multiprofessional teams managing patients with cancer will be required to be confident in the use of AI in their clinical practice, whether they are using it directly, such as in the setting of risk stratification tools for patient management decisions, or acting upon diagnostic information which has been assisted by AI in the context of a histology or imaging report. They will need to understand the perspectives of their patients in relation to the use of AI and how this may impact acceptance of a diagnosis or management plan. Patients also need to feel confident and comfortable with the potential for AI input into their care.

The literature to date is, however, limited in relation to such key stakeholder attitudes to the use of AI in clinical practice^19^ despite evidence that the perceptions and attitudes of potential users of AI in health care—the clinicians, patients, health‐care providers, and policymakers—will be important in influencing its adoption^20^, ^21^, ^22^, ^23^.

Specifically in relation to the role of AI in histopathological diagnosis where these technologies are already being used in clinical practice, the evidence base around important issues such as the impact of AI on pathologist behavior and on patient satisfaction is currently limited^12^, which is reflective of the early stages of adoption. The literature is even more limited in respect to the patient viewpoint on AI‐assisted pathological diagnosis^24^, ^25^, ^26^, and studies are lacking in relation to the downstream acceptance of AI‐assisted diagnostic reports among clinical team members directly managing patients and on related issues such as whether that information needs to be conveyed within the pathology report.

The aim of this research was therefore to explore the opinions and attitudes of both patients and the multidisciplinary clinical team who have experience in managing patients with prostate cancer (or who are undergoing investigation for potential prostate cancer) related to the use of AI in assisting pathological diagnosis, and specifically in terms of the clinical group, to assess opinion related to their trust in AI used by other members of the clinical team (pathologists), which is a novel and important question.

## 2 METHODS

### 2.1 Study design

In the absence of existing validated surveys/questionnaires suitable and specific enough to allow us to explore the subject of AI‐assisted pathological diagnosis in the setting of prostate biopsies, we developed novel and complementary cross‐sectional surveys for patients and clinical teams in order to understand better the perspectives of the two groups, and in a way that would allow the exploration of the similarities and differences in these perspectives. The former was for patient members of prostate cancer support groups within the United Kingdom, and the latter for non‐pathologist multidisciplinary clinical team members (hereafter ‘Clinicians’) managing patients with prostate cancer (or who are undergoing investigation for potential prostate cancer) within the Prostate Cancer Pathway at a large academic teaching hospital.

The questions for this were developed by AG and LB and were informed by discussions among a group of histopathologists with experience in using digital pathology and in evaluating digital pathology and prostate biopsy diagnostic assistance AI as standard of care (AG, LB, RC, CV). The question development was contributed to by patient members of a prostate cancer support group (RS, JC, ES), and available literature on the topic of AI in health care, including our own prior work which examined more broadly patient attitudes to digital pathology and AI^24^. The pathologists in the study were based in a histopathology department that has been 100% digital for surgical pathology slides since 2020 (Philips digital pathology system) and have also been involved in the evaluation of Paige Prostate AI as part of the Articulate Pro study^27^. Guidance available from the literature was used to ensure the quality of the surveys^28^, ^29^. While a pre‐survey pilot was not formally conducted, the survey questions were re‐reviewed by the patient representatives (RS, JC, ES) to provide assurance that the content and wording would be understandable and acceptable to the target patient group. The participant information included an outline of the potential role of AI for diagnostic assistance in histopathology, and for the patient group included a basic outline of the role of pathology in the reporting of prostate biopsies, with links to external online resources for further information. Because of the novelty of the developed surveys, formal external validation was not feasible, but the analysis of the results includes comparison of our findings with the limited existing literature on related topics.

The study received Ethics Approval from the University of Oxford Central University Research Ethics Committee (CUREC, CUREC Approval Reference: R90063/RE001) and Approval from the Health and Research Authority (HRA) and Health and Care Research Wales (IRAS project ID 333795, REC reference 23/HRA/5084), with subsequent hospital trust management approval (May 2024).

The surveys to clinicians and to Prostate Cancer Support Groups (patient group) included generic questions related to diversity data: age, nationality and ethnicity, sex and gender, and, additionally for the patient group, geographical location. There followed 10 questions for the clinician survey and 13 questions for the patient survey, the themes of which overlapped for certain topics. The themes included use of AI for prostate biopsy diagnosis including questions around acceptance and confidence in AI, privacy and transparency, patient expectations, education related to the use of AI, and questions focussed on patient access to histopathology reports and what should be included/recorded within that report. For the clinician group, there were additional questions exploring the current level of experience of the utility of AI in health care and general attitudes to this.

There was an opportunity for free text comments.

The survey questions are available as Supporting Information.

### 2.2 Survey distribution

The anonymised survey was hosted on the Jisc online survey platform. The consent process was integrated with the participant information presented at the start of the survey. The participants were informed that they could withdraw their participation at any stage during the survey by closing the web browser. The participants were required to provide consent and to confirm an age of 18 years or over before the survey questions were launched.

The survey to patients was open for 6 weeks from 6 February 2024, and that for clinicians was open for one calendar month from 22 May 2024. A reminder email was sent 2 weeks after the surveys opened, but no further contact was made thereafter.

#### 2.2.1 Patient survey

A scoping email was sent to the designated contact individual for 32 prostate cancer support groups across the UK, which were identified from the Tackle Prostate Cancer website (https://tackleprostate.org/), to determine their willingness to participate in the study. Of the groups contacted, 12 responded and agreed to distribute the details of the survey via email through the designated individual or group lead to their members, and were thus included. These groups were based in Wales, London and the South East, South, South West, East, North East, and North West of England. It is not possible to exclude that some patients could have been members of more than one group.

#### 2.2.2 Clinical team

The survey was distributed by email to confirmed oncologists, radiologists, urologists or specialist nurse members of the clinical team managing patients within the prostate cancer pathway at the designated hospital.

### 2.3 Data analysis

For the purposes of statistical analysis of the survey responses, the responses, each recorded as a 6‐point Likert scale, have been reported as ordinal variables with a range from *strongly disagree* to *strongly agree*. The data were dichotomised to calculate the percentage of *agree* or *strongly agree* responses among all replies, with 95% confidence limits calculated for this proportion. Free text responses in relation to awareness and attitudes to the potential for AI‐assisted diagnosis in prostate biopsy interpretation (questions 8–13) were reviewed and grouped into broad themes for the purpose of qualitative analysis.

## 3 RESULTS

### 3.1 Survey response rates

The patient survey was distributed via 12 prostate cancer support groups to at least 1092 group members (numbers provided by five groups; number of members contacted for the remaining seven groups is not known, and therefore the percentage response rate cannot be provided). There were 130 responses (including three made on behalf of a patient). Occasional questions were not answered in full by all respondents (denominators indicated in the presented results). Forty free text comments were received in relation to AI, four of which were non‐specifically related to the survey or to the patient's personal health ‘journey’ and which were not further analysed. The remaining 36 comments were grouped into broad themes, with some comments covering more than one theme (Table 1). Additional selected comments are presented within the following sections.

**TABLE 1:** Summary of themes from 36 free text comments from the patient group related to the questions on AI.

Theme	Number of comments	Example quotes
Optimism around the potential for AI in this setting or in health care generally	22/36	*‘Anything that can improve the diagnostic process and especially cutting down the time associated with the process must be of the utmost importance and should be pursued vigorously.’* *‘AI is a developing technology and clearly has applications in medical science and diagnosis. A computer never gets tired, bored or lazy. Reducing variables in diagnosis is important.’* *‘As I understand at the moment AI would enhance diagnosis by accessing a knowledge pool in order to compare results with known results, this can only enhance accuracy in deciding potential treatment options and likely outcomes. All to the better. It seems to me that any potential to compare past biopsy with subsequent ones in patient may be crucial in recognizing change; which I believe is vital. I would feel confident in this being more accurate than a pathologist alone.’* *‘The future lies with AI which in time will accelerate the speed and quality of treatment. The sooner the better.’*
Trust in the use of AI in this setting	19/36	
Personal need for confidence in the technology	4/36	*‘Confidence in the integrity of the scientific reliability of the use of AI as a tool is the key.’*
The need for quality control/human/pathologist oversight of the diagnosis assisted by AI	11/36	*‘I think it is important that the NHS employs every tool at its disposal in order to improve both the efficiency and accuracy of diagnosis, but human pathologists should still be required to validate the results of AI‐assisted diagnosis and seek a second pathologist's opinion if in doubt.’* *‘AI is the future but must always be used in conjunction with a qualified professional in whatever field of medicine it is aimed at’*. *‘I think that anything which helps to improve the accuracy and speed of a prostate cancer diagnosis (including AI) is very welcome but would always like a human being to also be involved in the process.’* *‘Although not a clinician nor a scientist I believe that increasing the use of AI will speed up the reporting of the results of innumerable tests in medicine. In principle a clear positive or clear negative result could be returned instantly. An unclear result may need to be checked by a trained pathologist but there is rarely 100% certainty about medical results.’* *‘When prostate cancer is diagnosed by PSA & subsequent tests, without any other significant symptoms, the patient needs to have total confidence in the diagnoses & those offering treatment. The symptoms & side effects of treatment can be much worse than those experienced before treatment started …’*
Concern over potential diagnostic errors/mistakes related to AI	7/36	*‘AI is only as good as the computer programmers. There is a danger of gradual shift into the belief that if AI says something it must be right resulting in errors and therefore diagnosis being missed.’* *‘… I would suggest that it would take time to develop confidence which also excludes consideration of “coverups” in the system for various reasons.’*

*Note*: Some of these comments included more than one theme.

The clinician survey was distributed to 29 individuals, with nine responses (31%). It is not possible to determine the clinical roles of those who responded; this was not asked in the survey to avoid the potential for inadvertent identification of a participant given the limited number of invitations. Two free text comments were received, one of which was non‐specific in relation to the survey questions.

### 3.2 Participant characteristics

Of the patient group, all participants were over 45 years old, and 86% were over 65 years old. All considered themselves to be male and were assigned male at birth. The clinician participants' age range was 25–64 years; 44% were male and 56% were female. See Table S1, which also details the place of residence of patient respondents.

### 3.3 Trust in the use of AI as a diagnostic assistance tool

While a pathologist was also involved in the diagnosis of a prostate biopsy, patients expressed high levels of trust in the use of AI assistance (Table 2). For example, 122 of 130 (94%) patients agreed or strongly agreed that they would be comfortable with AI‐assisted diagnosis in the interpretation of a prostate biopsy, provided that a pathologist retained responsibility for the final diagnostic report (currently AI workflows in clinical use involve a pathologist with the AI).

**TABLE 2:** Patient trust in the use of artificial intelligence (AI) as a diagnostic assistance tool.

	Strongly disagree	Disagree	Neutral	Agree	Strongly agree	Do not know	Number of respondents	Proportion with reply of agree or strongly agree (95% CI)
I would be comfortable with the idea of AI‐assisted diagnosis in the interpretation of my prostate biopsy but would want to know that a pathologist was responsible for the interpretation of the AI output and the final diagnostic report	2	1	5	40	82	0	130	122/130 (93.8%) (88.2–97.3%)
I would feel comfortable with an AI diagnosis that my biopsy was benign (no cancer) but would want a pathologist to double check if this result was accurate	2	3	4	50	70	0	129	120/129 (93.0%) (87.2–96.8%)
I feel that it is the responsibility of the clinician (pathologist) to make the decision as to whether AI assistance is appropriate in the diagnosis of my biopsy	4	9	11	65	38	1	128	103/128 (80.5%) (72.5–86.9%)
If available, I would prefer that AI assistance is always used for diagnosing prostate cancer	5	7	39	50	29	0	130	79/130 (60.8%) (51.8–69.2%)
I would be comfortable with the idea that AI‐assisted interpretation of my prostate biopsy might replace a second opinion from another pathologist	14	24	28	48	13	2	129	61/129 (47.3%) (38.4–56.3%)
I would want to be able to decide whether AI assistance was used in the diagnosis of my biopsy	9	36	47	20	14	3	129	34/129 (26.4%) (19.0–34.8%)
I would prefer that AI assistance is not used without my explicit consent	23	50	27	17	10	3	130	27/130 (20.8%) (14.2–28.8%)
I would feel comfortable with an AI diagnosis that my biopsy was benign (no cancer) *without* a pathologist checking that this result was accurate, if the pathologist was confident in the AI output	34	47	16	23	7	3	130	30/130 (23.1%) (16.1–31.3%)
I am concerned about the privacy of my data if AI assistance is used	35	41	34	11	5	3	129	16/129 (12.4%) (7.3–19.4%)
I would prefer that all steps in my diagnosis were performed by a human without AI assistance	32	59	25	5	7	1	129	12/129 (9.3%) (4.8–15.7%)

*Note*: Respondents were given the option to omit responses for questions; hence, the number of responses was not always the maximum of 130.

By comparison, patient agreement was less regarding the concept of a more autonomous use of AI in relation to a diagnosis (Table 2) with 30/130 (23%) of patients agreeing or strongly agreeing that they would be comfortable accepting an AI diagnosis of benign in the absence of pathologist verification (in the circumstance that the pathologist was confident in the AI output), and 61/129 (47.3%) agreeing or strongly agreeing that they would be comfortable with the scenario of AI acting in replacement of a bona fide second opinion from another pathologist.

The issue of trust in a diagnosis assisted by AI was a recurrent theme in the free text comments from the patient group (Table 1).

Although no seemingly definitively ‘anti‐AI’ comments were received, free text comments from two patients relay a degree of caution about the use of AI in this setting;

*‘There would need to be strong safeguards against possibility of ‘glitches' in the software leading to poor diagnoses.’*

*‘I have a need to understand how the technology works and how it is better than an experienced person. There is a fear that this will just enable a ‘*health service’ *to dumb down the level of people dealing with biopsies and rely on an IT input which may not be correct.’*

While sample size was more limited, the clinician responses convey an indication of general agreement between patients and clinicians regarding trust in the use of AI as a diagnostic assistance tool (Table S2). For example, 9/9 (100%) of clinicians agreed or strongly agreed that they would be comfortable with AI‐assisted diagnosis in the interpretation of a prostate biopsy provided a pathologist retained responsibility for the final diagnostic report. Similar to the patient group, around half (5/9, 55.6%) expressed comfort with the idea that AI‐assisted interpretation of a prostate biopsy might replace a second opinion from another pathologist, with an indication of slightly greater (55.6%, 5/9) clinician comfort with an AI diagnosis that a prostate biopsy was benign (no cancer) without a pathologist checking the result.

In relation to whether AI assistance is appropriate in the diagnosis of a prostate biopsy, the patient group responses indicate a preference that the pathologist retains the responsibility as to whether AI is used (103/128, 80.5%). A similar response rate was seen from the clinicians (8/9, 88.9%), echoed in a free text comment from a clinical participant;

*‘Presuming there is appropriate governance around utilising AI, given that we know and trust the pathologists currently diagnosing prostate cancer I would be confident* (in the use of AI) *if they are confident.’*

### 3.4 Factors impacting potential comfort and acceptance of AI in diagnosis

The participants were asked about the impact of information about the use of AI‐assisted diagnosis on their level of comfort related to its use. Fifty‐seven of 130 (43.8%) patients suggested that they would be comfortable with the use of AI in this setting if it were clinically approved, without needing additional information about it, whereas 66/130 (50.8%) would like to have access to additional information. Only 5/130 (4%) of patients suggested that they would not want AI to be used, regardless of any additional information provided to them. The responses from the clinician group indicate perhaps a slightly greater desire for additional information (6/9, 67%).

**FIGURE 1:**
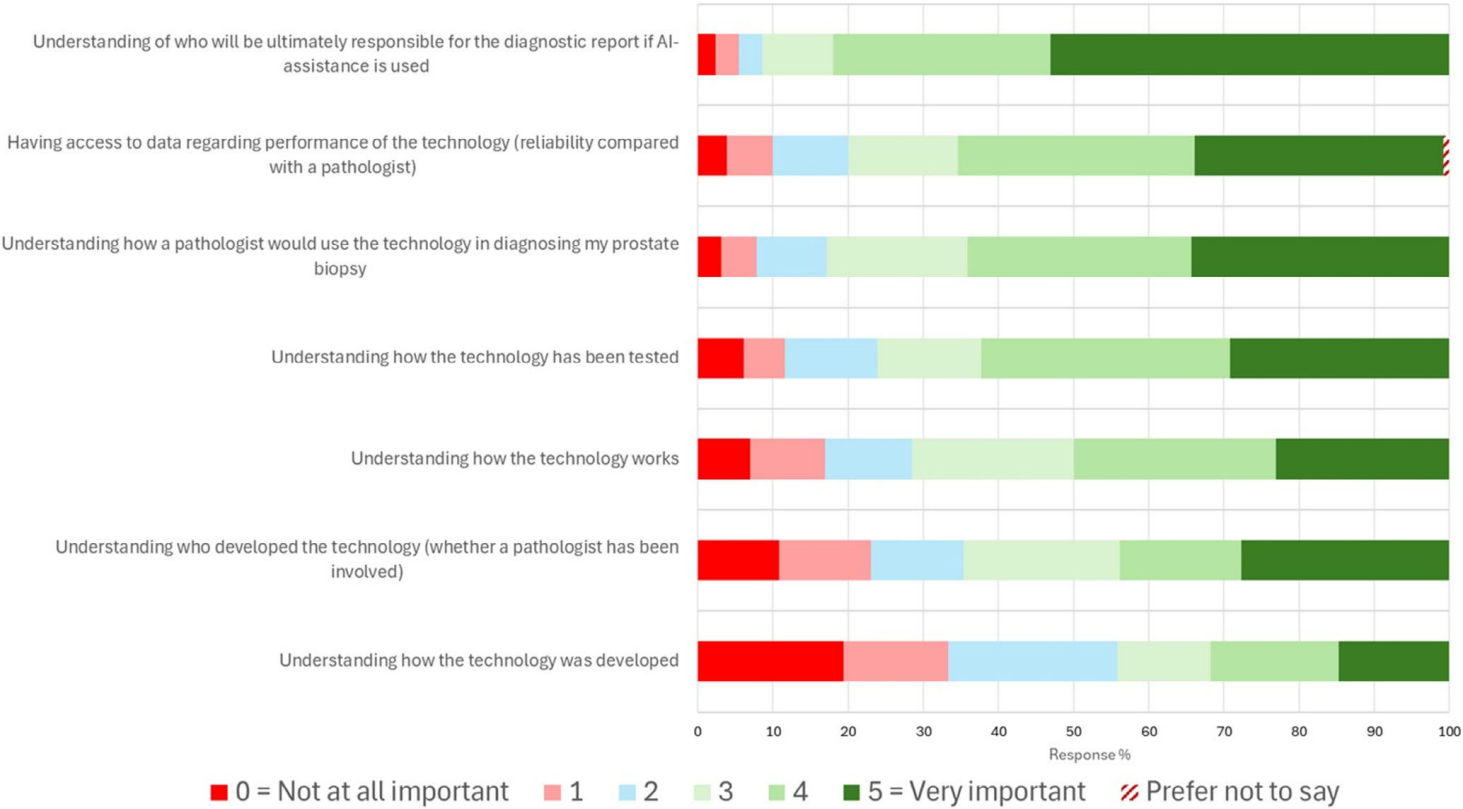
Factors that might impact patient acceptance of the use of AI‐assisted diagnosis for prostate biopsy reporting, and their perceived importance.

The importance of specific factors in the potential acceptance of the patient group to the use of AI in this setting is presented in Figure 1. Of these factors, how the technology had been tested and how it performed in relation to a pathologist were generally considered to be more important than understanding how the technology was developed. Understanding who retained ultimate responsibility for an AI‐assisted diagnosis was the factor most frequently reported as being ‘important’ or ‘very important’ by patients (105/128, 82.0%) and clinicians (9/9, 100%). The results for the clinician group are presented in Table S3.

### 3.5 Aspirations related to potential benefits of AI‐assisted diagnosis

The patients generally agreed that AI assistance might improve the efficiency of pathologists, reduce waiting times for prostate cancer diagnosis and improve the accuracy of a prostate cancer diagnosis, with 83.8% (109/130), 76.1% (99/130) and 84.6% (110/130), respectively, agreeing or strongly agreeing with these points. Example free text comments expanding on the aspirations of patients in relation to the use of AI are shown in Table 1.

The results from the clinician group indicate potentially similar opinions in relation to improved efficiency and reduced waiting times with AI, with 66.7% (6/9) agreeing or strongly agreeing; however, their aspirations would seem to be potentially more guarded around the prospect for AI assistance to improve the accuracy of diagnosis, with 55.6% (5/9) agreeing or strongly agreeing to this point.

The patient group had a tendency to believe that AI assistance would improve the confidence of both a patient and a doctor in the diagnostic report, with 66.2% (86/130) and 84.6% (110/130) agreeing or strongly agreeing with these points. While the number of respondents was small, the opinion of the clinician group on these points would seem to be potentially less optimistic: 11.1% (1/9) and 22.2% (2/9) agreeing.

The patients were also asked about their views on the development of AI in relation to pathology. Forty percent (52/130) suggested they are interested in understanding more about how AI technology is developed in this field, with 46.9% (61/130) suggesting that they would consider being personally involved in the development of AI. Ten percent (13/130) suggested they would not be.

### 3.6 Transparency in relation to reporting of biopsies which have had an AI‐assisted diagnosis

**FIGURE 2:**
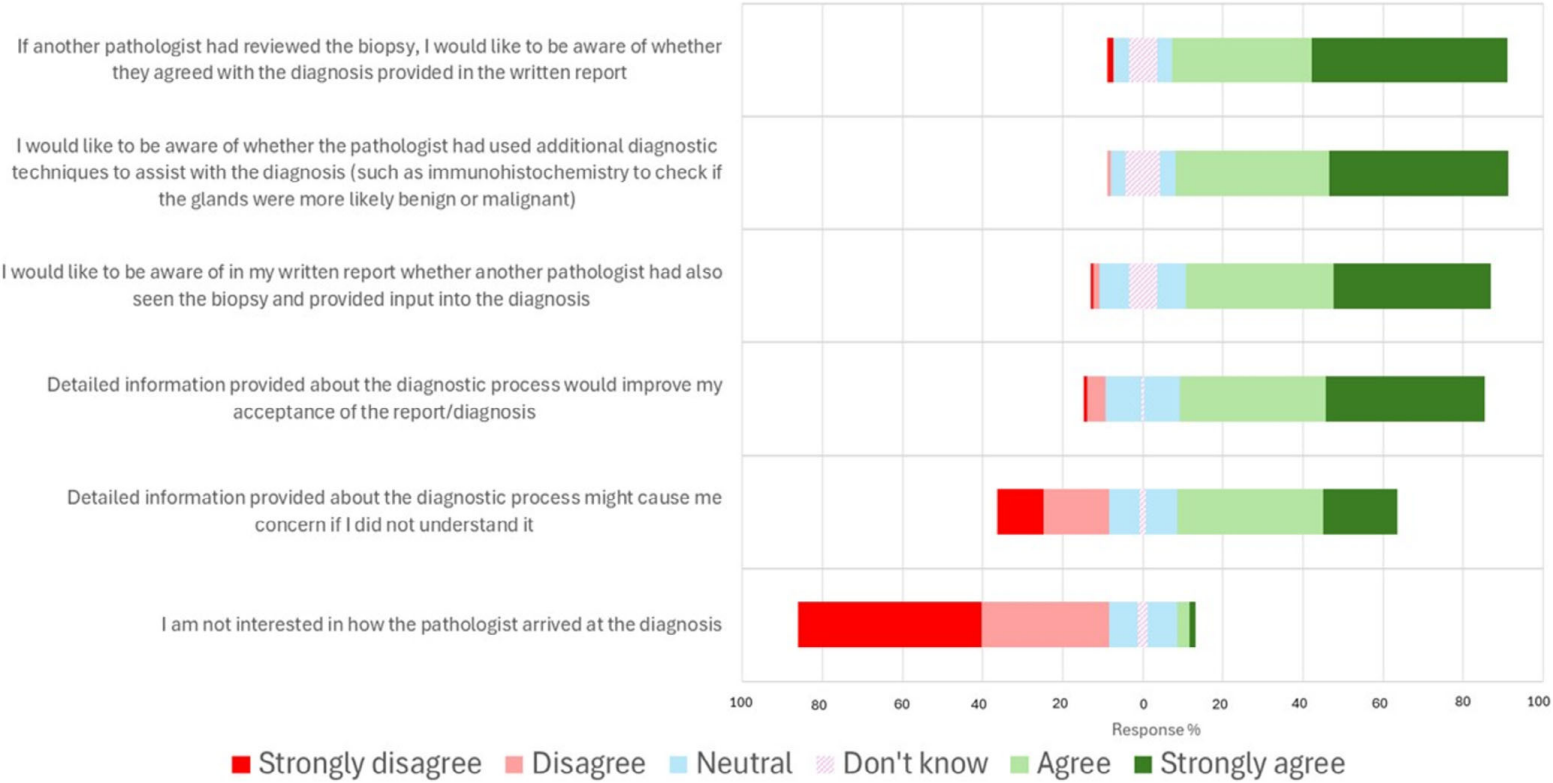
Patient responses regarding the content of the written diagnostic report in relation to reaching a diagnosis.

The perspectives of patient participants in relation to the content of the diagnostic histology report, both generally in reaching a diagnosis and specifically in relation to recording of AI output, were explored in detail (Figure 2). Significantly, all respondents in the patient group considered that a record of AI‐assistance output should be kept, and 84.6% (110/130) agreed that all details of the output should be recorded, with only 14.6% (19/130) suggesting that only the human interpretation of the AI output was required.

By contrast 83.1% (108/130) of patients agreed that a diagnostic report should include a record of the use of additional diagnostic techniques such as immunohistochemistry, and 76.2% (99/130) agreed that the report should record whether another pathologist has seen and provided input into the diagnosis, with 83.7% (108/129) wishing also to know whether that pathologist agreed with the diagnosis provided. For context, the participants were asked for additional detail in relation to their experience of personal receipt of the pathology report for their prostate biopsy (patients) or provision of that report to a patient (Table S4).

Of the clinician respondents, all agreed that a record of AI‐assistance output should be kept and favoured that all details of the AI output should be recorded.

### 3.7 Data protection and trustworthiness

In relation to data privacy and the use of AI, 12.4% of patients (16/129) expressed concern regarding the privacy of their data, although conversely 58.9% (76/129) disagreed that they had concerns related to this issue. Furthermore, the issue of data protection did not appear specifically within free text comments, although there were comments in relation to the trustworthiness of information technology (IT) and AI in general (Table 1), some focussing on media‐worthy events.

The responses from the clinician group would appear to indicate similar opinions in relation to patient data privacy, with 22.2% (2/11) agreeing and 55.6% (5/9) disagreeing that they had concerns.

### 3.8 Prior experience of AI in clinical practice

**FIGURE 3:**
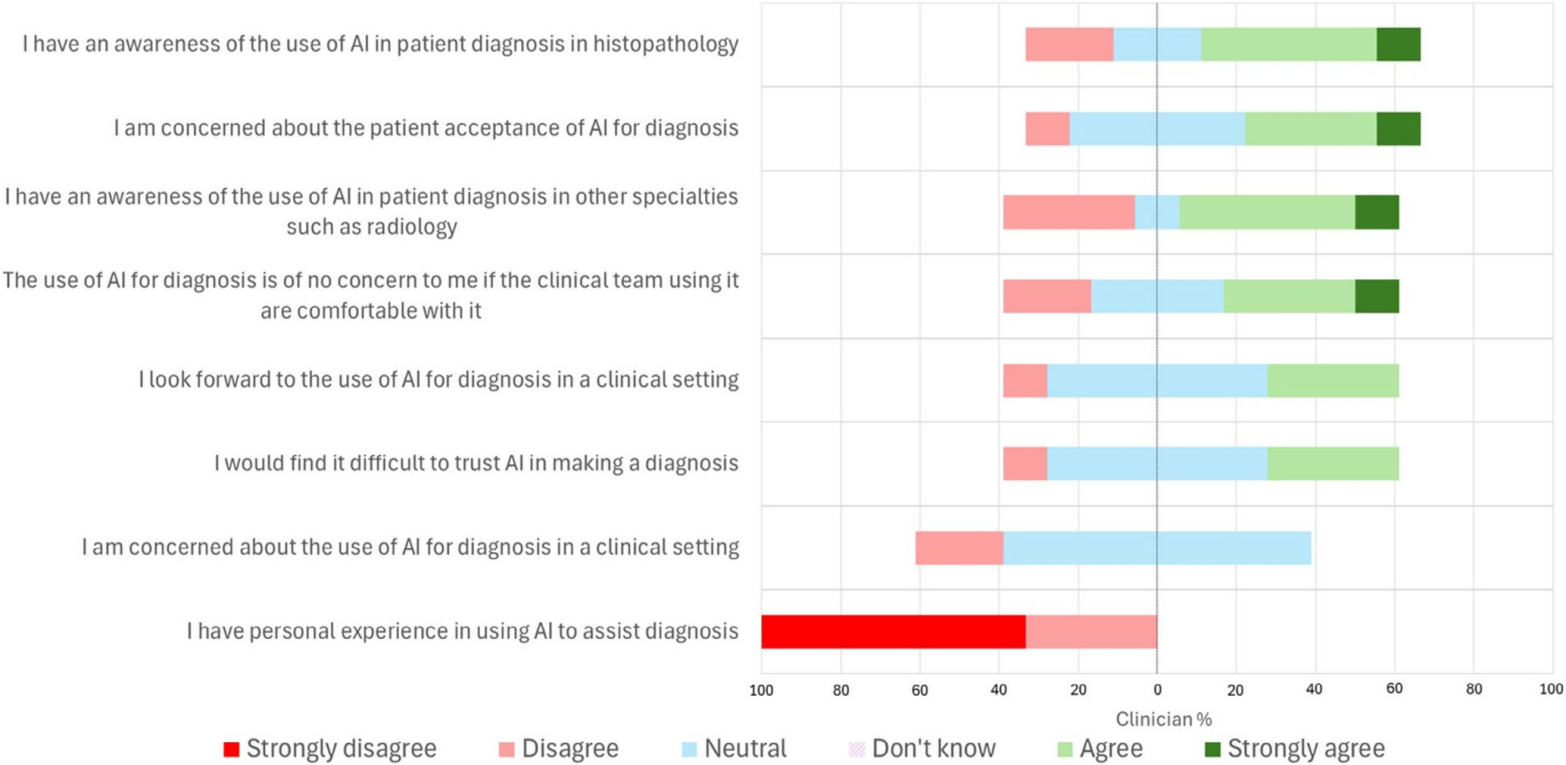
Clinician experience of AI in clinical practice and general opinions in relation to this.

The clinical participants were asked about current personal knowledge and experience of AI for diagnosis in health care (Figure 3). Around half (5/9, 55.6%) were aware of the use of AI in diagnostics (pathology and radiology); however, none had personal experience in this. Around 33.3% (3/9) suggested that they would find it difficult to trust AI in making a diagnosis, although 44.4% (4/9) suggested they would not be concerned about the use of AI if the clinicians using it were comfortable (with the AI), with 33.3% (3/9) remaining neutral on this point.

## 4 DISCUSSION

As AI now becomes a reality within specific aspects of health care, beyond its validation to ensure safety in clinical practice, it is essential to understand the real‐world acceptance of its utility among clinical teams working with it, and the patients for whom AI may be part of the diagnostic work‐up or clinical decision‐making.

While we acknowledge that the number of responses from the clinical group in our study was more limited than our patient group, we do feel that these responses are an important indication of potential opinion among this stakeholder group, and interestingly note that generally the responses in relation to trust in AI were similar to the patient group. Encouragingly, our results indicate a high potential acceptance of AI for diagnostic assistance in histological reporting, with only 4% of the patients suggesting that they would not want AI to be used (in this setting), and universal acceptance of the use of AI in this setting among our clinical group. However, and as evidenced by others,^19^, ^25^, ^26^, ^30^, ^31^ the confidence and acceptance of AI is impacted by having a ‘human‐in‐the‐loop’, in this case a pathologist retaining oversight and responsibility for the final diagnostic report. Where a pathologist retains such responsibility, 94% of the patients and 100% of clinicians in our study expressed comfort in the use of AI.

The comfort level appeared comparatively lower for an AI‐only benign (no cancer) diagnosis, with only 23% of patients expressing comfort in this scenario where a pathologist does not ‘check’ the result. There was a suggestion also of a degree of apprehension among the clinicians, 56% of whom expressed comfort in relation to this scenario. We asked this specifically because, under current practice, AI workflows still require a pathologist to review the histological findings and retain responsibility for final diagnosis; one of the future anticipated advantages of AI in the diagnostic setting is its potential value in efficiency gains, which could include ‘screening’ out biopsies that are considered by AI to be benign without further pathologist input, thereby freeing over‐stretched pathologist time for other potentially more complex cases.

While literature on this topic has to date often examined the viewpoint of the public more often than that specifically of patients, in a recent study, Rodler et al.^26^ sought the prospective views of patients in an academic centre in Munich undergoing radical prostatectomy for histologically confirmed prostate cancer, or MRI or biopsy investigation for suspected prostate cancer.^26^ They reported that ‘patients’ trust in AI was generally high but low when physicians were not checking results generated by an AI model, and that patient preference would be for AI‐assisted physicians (as opposed to physician or AI alone).

Our results are, therefore, reflective of the limited literature on this topic for the patient group, and our study indicates that clinicians may express similar levels of comfort/trust to the patients in relation to an AI‐assisted diagnostic report.

Important also is an understanding of the impact of an AI‐assisted diagnosis on patient acceptance of a treatment recommendation based upon that diagnosis, and similarly the willingness of a clinician to make a treatment recommendation based upon an AI‐assisted report. The evidence in relation to this is currently very limited and is specific to the patient group, and it necessarily relates to hypothetical scenarios given the early stages of AI implementation. However, some studies suggest that acceptance is higher when a clinician remains central to the diagnosis—whether with or without AI, compared with AI alone.^30^ Furthermore, the perceived risk/severity of the diagnosis has also been shown to influence how accepting patients/potential patients are of an AI‐assisted diagnosis.^26^, ^31^, ^32^, ^33^ To the best of the authors' knowledge, there are no studies to date which have examined the viewpoint of the clinician receiving an AI‐assisted diagnostic report. However, our results indicate the apparent willingness of the clinical group to accept the judgement of their pathologist colleagues as to whether it is appropriate to use AI, 89% of clinicians considering that a pathologist should retain responsibility for the decision as to whether AI assistance is appropriate, with 77% being in agreement or of neutral opinion that the use of AI for diagnosis is of no concern if the clinician (in this case pathologist) using it is comfortable. This is a key point of consideration, not only because it is important for all members of a clinical team to trust the diagnostic report upon which management decisions are made but also because this clinical trust has potential impact on patient acceptance of such a recommendation. Interestingly, our survey results indicate that in relation to the potential for AI assistance to improve the levels of confidence in a diagnostic report among both patients and clinicians, the patient group felt confident that it would (67% and 84% for the patient and clinician confidence, respectively).

This wider concept was discussed in a recent review of stakeholder perspectives on AI^19^ which reports that generally clinicians are seen as ‘intermediaries’ between patients and AI, their endorsement of AI being key to patient trust (in the AI). This was noted by Lyso et al.^25^ in their focus group‐based work looking at the expectations of men in relation to the use of AI in the diagnostic process for prostate cancer (in the context of MRI). They noted that ‘participants’ trust in AI was not in the envisioned technology per se, but rather in the health‐care system and in the doctors who, they believed, would only employ well‐tested, beneficial tools.

Regarding additional expectations around the use of AI, the viewpoints in our study of both patient and clinical groups would seem to be aligned in relation to the anticipated benefit of AI in reducing waiting times for diagnosis (76% vs 67%), likely related to perceived potential for improved pathologist efficiency. Overall, and as seen by others, there was much positivity expressed by patients in the anticipation of the benefits of AI for their own care and within the wider National Health Service (NHS).

Our study indicates that transparency is important in relation to the use of AI in this setting with the preference of the patient group (100% of respondents) being that the use of AI for diagnostic assistance is declared within the pathology report. Ethical and legal issues around AI in health care are written about increasingly as AI begins to become reality,^34^, ^35^, ^36^ although very few studies have specifically addressed this issue among patient groups and members of the public.^19^, ^37^ These are issues that will need continued consideration as we move towards the reality of wider AI integration within health care but equally should not impede the implementation of approved technology which can benefit patient care. Indeed, stakeholders have expressed caution in relation to an ‘exceptional approach’ to the use of AI in contrast to other ‘sophisticated technologies’ around disproportionate requirements for patient information and choice^35^; by comparison, a pathologist is not ‘required’ to disclose the use of immunohistochemistry in reaching a diagnosis nor informal consultation with a colleague. Appropriate guidance on these matters is currently lacking and will be welcomed as AI becomes a more integral part of the diagnostic pathway. These issues are often poorly understood by clinical teams, as a recent study examining this topic in relation to digital pathology has shown,^38^ and education on such aspects of practice will be important.^12^ In a setting where AI is being used for diagnostic assistance, urologists and other non‐pathologist members of the clinical team should be made aware of its use and be appropriately resourced to understand its role and thus confident in responding to patient enquiries should they arise. Information can also be provided and made accessible to patients more generally by hospitals, for example, through provision of patient information leaflets in relation to technologies in use.

Moreover, education in general is seen as being integral to the acceptance and implementation of AI in health care. This can be addressed through education within a specific setting, in this case prostate biopsy diagnosis, but there would be benefit in more generic educational initiatives for the benefit of patients and health‐care workers, augmented through collaborations with charitable organisations with the ability to reach out to patients and their supporters. Furthermore, as AI becomes a reality in health care, reporting of the experience of its ‘users’ will be essential in order to build trust among clinicians and patients alike. Our study highlights interest in the provision of information related to the development and ‘testing’ (validation) of AI as well as its reliability in comparison to a clinician (in this case pathologist), and indicates that understanding of these topics may improve the likelihood of acceptance of the technology, at least among a proportion of people. The notion that patients are interested in educational material in relation to AI in health care is consistent with our previous research with patient supporters of Prostate Cancer UK,^24^ and it was also encouraging to note that almost half of our survey respondents would consider future personal involvement in the development of AI which could include the development of patient‐appropriate information resources. The involvement of patients in the development of AI and in its clinical implementation has a significant role in its successful adoption, and we would advocate that this should be an integral part of future studies in this regard.

### 4.1 Limitations

We acknowledge the limited number of respondents to the clinical team survey; however, we had a good response rate to the survey from this group and a complete dataset from each respondent and are thus confident in the data being illustrative of a perspective from this important stakeholder group which is worthy of further exploration. The findings are an important consideration in these early stages of adoption of AI and set a benchmark for future studies, highlighting areas of potential focus which could also include perspectives on novel AI in the context of biological insight/risk prediction in prostate cancer.

The survey did not explore in depth the potential impact on the responses of participant characteristics such as educational background or competence/confidence in technology. However, it has been noted in a recent review that commonalities exist between the view of those with and without direct experience of AI;^19^ and therefore, we feel that the findings of our study remain valid. Furthermore, for the purpose of scene‐setting and in acknowledgment of the potential limited understanding of the subject matter, a baseline basic overview of the role of pathology in prostate biopsy reporting and the potential for AI in this setting were provided to participants in the pre‐consent information.

We acknowledge that we have not sought the prospective opinion of men undergoing prostate biopsy as to how they would feel about AI being used in the provision of a diagnosis. It is noted, however, that the few studies which have approached patients in this situation have reported similar views to those that we have shown.^25^, ^26^ We also accept that it is likely that the majority of men completing the patient survey would have had a malignant diagnosis, and it is possible that the responses of men with a benign diagnosis for their prostate biopsy may have differed.

## 5 CONCLUSIONS

We have shown that there is much positive anticipation for the potential benefits associated with the use of AI in the diagnostic setting for prostate cancer. While there is a high level of acceptance of AI in this role, it is evident that this is impacted by and dependent upon retention of clinician oversight of the AI. This is concordant with the available literature and reflects the current real‐world application of AI in this diagnostic setting, which is limited to its utility as a diagnostic‐assistance aide for a pathologist to use at their discretion.

To the best of the authors' knowledge, this is the first study to specifically consider the perspective of patient‐facing clinicians and their opinions in relation to AI‐assisted diagnostic reports upon which their patient management decisions will be made. This is an important perspective to understand as we now move forward with real‐world AI applications in urological practice and the wider health‐care setting, and future studies looking beyond hypothetical scenarios of AI use will be welcome.

## AUTHOR CONTRIBUTIONS

**Lisa Browning:** Conceptualization; methodology; validation; formal analysis; investigation; data curation; writing—original draft; writing—review and editing; visualization; supervision. **Abhisek Ghosh:** Conceptualization; methodology; validation; formal analysis; investigation; data curation; writing—original draft; writing—review and editing; visualization; supervision. **Monica Dolton:** Project administration. **Kate Hutton:** Investigation; data curation. **Jacqueline Birks:** Formal analysis. **Richard Scheffer:** Methodology. **Ewart Stanislaus:** Methodology. **James Crofts:** Methodology. **Richard Colling:** Writing—review and editing. **Richard Bryant:** Writing—review and editing. **Clare Verrill:** Conceptualization; methodology; writing—original draft; writing—review and editing; supervision; funding acquisition.

## CONFLICT OF INTEREST STATEMENT

The authors confirm that there are no conflicts of interest to declare.

## Supporting information


**Table S1** Participant demographic details.

**Table S2** Clinician trust in the use of artificial intelligence (AI) as a diagnostic assistance tool.

**Table S3** Factors that might impact clinician acceptance of the use of AI‐assisted diagnosis for prostate biopsy reporting, and their perceived importance.

**Table S4** Participant experience related to receipt of a histology report for their prostate biopsy (patient opinion) or provision of a diagnostic report to a patient (clinician opinion).

**Data S1.** A Survey to Investigate Clinician Attitudes towards Artificial Intelligence‐assisted Prostate Cancer Diagnosis.

**Data S2.** A Survey to Investigate Patient Attitudes towards Artificial Intelligence‐assisted Prostate Cancer Diagnosis.
