# Supplementary material for: Exploring patient and clinician opinions, perspectives and acceptance of the use of artificial intelligence in the histological diagnosis of prostate cancer
Source: BJUI Compass. 2025 Nov 9;6(11):e70108. doi: 10.1002/bco2.70108 (PMC12598096; doi:10.1002/bco2.70108)
Supplement: Supplementary file 1 — Table S1 Participant demographic details. [file BCO2-6-e70108-s006.docx]

**SUPPLEMENTAL TABLE S1**

Participant demographic details.

| Age % | Patients (n=130) | Clinicians (n=9) |
| --- | --- | --- |
| 18-24 | 0.0 | 0.0 |
| 25-34 | 0.0 | 11.1 |
| 35-44 | 0.0 | 33.3 |
| 45-54 | 0.8 | 22.2 |
| 55-64 | 13.1 | 22.2 |
| 65-74 | 46.9 | 0.0 |
| 75 years and over | 39.2 | 0.0 |
| Prefer not to say | 0.0 | 11.1 |
| **Sex and gender %** |  |  |
| Female | 0.0 | 55.6 |
| Male | 100.0 | 44.4 |
| Prefer not to say | 0.0 | 0.0 |
| **National identity %** |  |  |
| British | 73.1 | 66.7 |
| English | 33.8 | 11.1 |
| Welsh | 2.3 | 0.0 |
| Scottish | 0.8 | 0.0 |
| Northern Irish | 0.0 | 0.0 |
| Other | 7.7 | 0.0 |
| Prefer not to say | 0.0 | 22.2 |
| **Place of residence %** |  |  |
| England – South West | 20.0 | - |
| England – South East | 50.0 | - |
| England – Greater London | 12.3 | - |
| England – East of England | 4.6 | - |
| England – Midlands | 1.5 | - |
| England – North East | 0.8 | - |
| England – North West | 7.7 | - |
| Scotland | 0.0 | - |
| Wales | 1.5 | - |
| Other | 1.5 | - |
| Prefer not to say | 0.0 | - |
