# Supplementary material for: Exploring patient and clinician opinions, perspectives and acceptance of the use of artificial intelligence in the histological diagnosis of prostate cancer
Source: BJUI Compass. 2025 Nov 9;6(11):e70108. doi: 10.1002/bco2.70108 (PMC12598096; doi:10.1002/bco2.70108)
Supplement: Supplementary file 2 — Table S2 Clinician trust in the use of artificial intelligence (AI) as a diagnostic assistance tool. [file BCO2-6-e70108-s004.docx]

|  | **Strongly disagree** | **Disagree** | **Neutral** | **Agree** | **Strongly agree** | **Don’t know** | **Number of respondents** | **Proportion with reply of agree or strongly agree**  **(95% CI)** |
| --- | --- | --- | --- | --- | --- | --- | --- | --- |
| I would be comfortable with the idea of AI-assisted diagnosis in the interpretation of a prostate biopsy, but would want to know that a pathologist was responsible for the interpretation of the AI output and the final diagnostic report | 0 | 0 | 0 | 5 | 4 | 0 | 9 | 9/9 (100%)  (66.4% - 100%) |
| I would feel comfortable with an AI diagnosis that a prostate biopsy was benign (no cancer), but would want a pathologist to double check this result was accurate | 1 | 0 | 3 | 4 | 1 | 0 | 9 | 5/9 (55.6%)  (21.2% - 86.3%) |
| I feel that it is the responsibility of the clinician (pathologist) to make the decision as to whether AI assistance is appropriate in the diagnosis of a prostate biopsy | 0 | 1 | 0 | 7 | 1 | 0 | 9 | 8/9 (88.9%)  (51.8% - 99.7%) |
| If available, I would prefer that AI assistance is always used for diagnosing prostate cancer | 0 | 2 | 5 | 2 | 0 | 0 | 9 | 2/9 (22.2%)  (2.8% - 60.0%) |
| I would be comfortable with the idea that AI-assisted interpretation of a prostate biopsy might replace a second opinion from another pathologist | 0 | 1 | 3 | 4 | 1 | 0 | 9 | 5/9 (55.6%)  (21.2% - 86.3%) |
| I think a patient would want to be able to decide whether AI assistance was used in the diagnosis of their biopsy | 1 | 2 | 3 | 2 | 1 | 0 | 9 | 3/9 (33.3%)  (7.5% - 70.1%) |
| I would prefer that AI assistance is not used without explicit patient consent | 1 | 2 | 4 | 1 | 1 | 0 | 9 | 2/9 (22.2%)  (2.8% - 60.0%) |
| I would feel comfortable with an AI diagnosis that a prostate biopsy was benign (no cancer) without a pathologist checking that this result was accurate, if the pathologist was confident in the AI output | 1 | 3 | 0 | 4 | 1 | 0 | 9 | 5/9 (55.6%)  (21.2% - 86.3%) |
| I am concerned about the privacy of patient data if AI assistance is used | 1 | 4 | 2 | 1 | 1 | 0 | 9 | 2/9 (22.2%)  (2.8% - 60.0%) |
| I would prefer that all steps in a prostate biopsy diagnosis were performed by a human without AI-assistance | 1 | 4 | 4 | 0 | 0 | 0 | 9 | 0/9 (0%)  (0% - 33.6%) |

**SUPPLEMENTAL TABLE S2**

Clinician trust in the use of artificial intelligence (AI) as a diagnostic assistance tool.
