# Supplementary material for: Exploring patient and clinician opinions, perspectives and acceptance of the use of artificial intelligence in the histological diagnosis of prostate cancer
Source: BJUI Compass. 2025 Nov 9;6(11):e70108. doi: 10.1002/bco2.70108 (PMC12598096; doi:10.1002/bco2.70108)
Supplement: Supplementary file 3 — Table S3 Factors that might impact clinician acceptance of the use of AI‐assisted diagnosis for prostate biopsy reporting, and their perceived importance. [file BCO2-6-e70108-s005.docx]

|  | **Not at all important**  **0** | **1** | **2** | **3** | **4** | **Very important**  **5** | **Number of respondents** | **Proportion with reply of important (4) or very important (5)**  **(95% CI)** |
| --- | --- | --- | --- | --- | --- | --- | --- | --- |
| Understanding how the technology works | 0%  (0/9) | 0%  (0/9) | 11%  (1/9) | 22%  (2/9) | 22%  (2/9) | 44%  (4/9) | 9 | 67% (6/9)  (29.9% - 92.5%) |
| Understanding how the technology was developed | 0%  (0/9) | 0%  (0/9) | 22%  (2/9) | 33%  (3/9) | 33%  (3/9) | 11%  (1/9) | 9 | 44% (4/9)  (13.7% - 78.8%) |
| Understanding who developed the technology (whether a pathologist has been involved) | 0%  (0/9) | 0%  (0/9) | 11%  (1/9) | 22%  (2/9) | 22%  (2/9) | 44%  (4/9) | 9 | 67% (6/9)  (29.9% - 92.5%) |
| Understanding how the technology has been tested | 0%  (0/9) | 0%  (0/9) | 0%  (0/9) | 11%  (1/9) | 44%  (4/9) | 44%  (4/9) | 9 | 89% (8/9)  (51.8% - 99.7%) |
| Having access to data regarding performance of the technology (reliability compared with a pathologist) | 0%  (0/9) | 0%  (0/9) | 0%  (0/9) | 22%  (2/9) | 22%  (2/9) | 56%  (5/9) | 9 | 78% (7/9)  (40.0% - 97.2%) |
| Understanding how a pathologist would use the technology in diagnosing a prostate biopsy | 0%  (0/9) | 0%  (0/9) | 0%  (0/9) | 11%  (1/9) | 44%  (4/9) | 44%  (4/9) | 9 | 89% (8/9)  (51.8% - 99.7%) |
| Understanding of who will be ultimately responsible for the diagnostic report if AI-assistance is used | 0%  (0/9) | 0%  (0/9) | 0%  (0/9) | 0%  (0/9) | 11%  (1/9) | 89%  (8/9) | 9 | 100% (9/9)  (66.4% - 100%) |

**SUPPLEMENTAL TABLE S3**

Factors that might impact clinician acceptance of the use of AI-assisted diagnosis for prostate biopsy reporting, and their perceived importance.
