# Supplementary material for: Exploring patient and clinician opinions, perspectives and acceptance of the use of artificial intelligence in the histological diagnosis of prostate cancer
Source: BJUI Compass. 2025 Nov 9;6(11):e70108. doi: 10.1002/bco2.70108 (PMC12598096; doi:10.1002/bco2.70108)
Supplement: Supplementary file 4 — Table S4 Participant experience related to receipt of a histology report for their prostate biopsy (patient opinion) or provision of a diagnostic report to a patient (clinician opinion). [file BCO2-6-e70108-s003.docx]

**SUPPLEMENTAL TABLE S4**

Participant experience related to receipt of a histology report for their prostate biopsy (patient opinion) or provision of a diagnostic report to a patient (clinician opinion).

| Did you receive a copy of your prostate biopsy pathology report? | Patient % (n=130) |
| --- | --- |
| Yes | 38.5 |
| I had details of the pathology report in a letter | 28.5 |
| I was given an opportunity to see the pathology report when I was given the result | 16.2 |
| I was provided with a copy of the pathology report as I requested this from the clinical team | 6.2 |
| I accessed the pathology report myself via the patient portal at my hospital Trust | 4.6 |
| I had access to the report via another means | 3.8 |
| No | 53.8 |
| Don’t know | 7.7 |
| Prefer not to say | 0.0 |

Of the clinicians surveyed, when asked if their patients received a copy of the pathology report; 7 stated No (77.8%); 1 stated Yes (11.1%); 1 stated Don’t know (11.1%).

| If you did not receive a copy of your prostate biopsy report, would you have liked a copy? | Patient % (of respondents to this question) |
| --- | --- |
| Yes | 48.4 |
| Yes (but only if I could discuss it with someone) | 42.1 |
| No | 2.1 |
| No preference | 7.4 |
| Don’t know | 0.0 |
| Prefer not to say | 0.0 |

| If you did have a copy of the pathology report, did you find this useful to you? | Patient % (of respondents to this question) |
| --- | --- |
| Useful | 86.3 |
| Neither useful nor not useful | 9.8 |
| Not useful | 0.0 |
| Don't know | 3.9 |

| Which of the following statements best applies regarding your understanding of the role of a histopathologist in making the diagnosis? | Patient % (n=130) |
| --- | --- |
| I have a clear understanding of the role of histopathologists in diagnosing prostate cancer | 46.2 |
| I am aware that a histopathologist is important in the diagnosis of prostate cancer, but I am unclear what role they play | 30.8 |
| I am unaware that histopathologists are important in the diagnosis of prostate cancer | 20.0 |
| I am not interested in how my prostate biopsy is diagnosed | 3.1 |
| Prefer not to say | 0.0 |
