# Supplementary material for: Exploring patient and clinician opinions, perspectives and acceptance of the use of artificial intelligence in the histological diagnosis of prostate cancer
Source: BJUI Compass. 2025 Nov 9;6(11):e70108. doi: 10.1002/bco2.70108 (PMC12598096; doi:10.1002/bco2.70108)
Supplement: Supplementary file 5 — Data S1. A Survey to Investigate Clinician Attitudes towards Artificial Intelligence‐assisted Prostate Cancer Diagnosis. [file BCO2-6-e70108-s002.pdf]

A Survey to Investigate Clinician Attitudes towards Artificial Intelligence-assisted Prostate Cancer Diagnosis

Questions

Please provide answers to the following Diversity Data questions. There is a 'prefer not to say' option for each.

Please select your age group:

**18-24 years**
**25-34 years**
**35-44 years**
**45-54 years**
**55-64 years**
**65-74 years**
**75 years or over**
**Prefer not to say**

National identity & ethnicity

How would you describe your national identity? (select all that apply)

**British**
**English**
**Welsh**
**Scottish**
**Northern Irish**
**Other (free text box to provide identity)**
**Prefer not to say**

What is your ethnic group? Choose one option that best describes your ethnic group or background.

**Asian or Asian British**	**Mixed or multiple ethnic groups (cont)**
**Bangladeshi**	**White and Asian**
**Chinese**	**White and black African**
**Indian**	**Any other mixed or multiple ethnic background (specify, if you wish)**
**Pakistani**	**White**
**Any other Asian background (specify, if you wish)**	**English, Welsh, Scottish, Northern Irish or British**
**Black, black British, Caribbean or African**	**Irish**
**African**	**Gypsy or Irish Traveller**
**Caribbean**	**Roma**
**Any other black, African or Caribbean background (specify, if you wish)**	**Any other white background (specify, if you wish)**
**Mixed or multiple ethnic groups**	**Other ethnic group**
**White and black Caribbean**	**Arab**
	**Any other ethnic group (specify, if you wish)**
	**Prefer not to say**

Sex & Gender

What sex were you assigned at birth? A question about gender identity will follow.

**Female**
**Male**
**Prefer not to say**

Which of the following best describes your gender?

**Male**
**Female**
**Non-binary or other (specify, if you wish)**

**Prefer not to say**

Questions 1 & 2 aim to explore the patient access to prostate biopsy pathology reports.

1. Do your patients receive a copy of the prostate biopsy pathology report?

Yes
No
Not applicable
Don't know
Prefer not to say

2. In relation to whether your patients receive a copy of the *written* prostate biopsy pathology report, please answer the following
(please tick all that apply);

The patients are routinely given an opportunity to see the pathology report when given the biopsy result
The patients are provided with a copy of the pathology report if this is requested by them
The patients are provided with details of the pathology report in a letter
The patients access the pathology reports themselves via the patient portal
The patients access the pathology reports by other means (please specify)
The pathology report is discussed with patients but they do not have the opportunity to see it
In my experience I do not feel that patients routinely want a copy of the pathology report
Not applicable
Don't know
Prefer not to say

FREE TEXT COMMENTS

Questions 3 & 4 aim to investigate what information may be important in relation to the diagnosis in the pathology report, and information that doctors should record.

Pathologists reporting prostate biopsies may ask another pathologist for their opinion if the findings are difficult to interpret (such as a very small amount of cancer), and they may also use several diagnostic techniques to arrive at the diagnosis. This is not always necessary.

3. Considering the process by which a pathologist arrived at a diagnosis for the prostate biopsy, please answer the following about what *you* would like to be made aware of in the written diagnostic report:

   a. Whether another pathologist had also seen the biopsy and provided input into the diagnosis.
   b. If another pathologist had reviewed the biopsy, whether they agreed with the diagnosis provided in the report.
   c. Whether the pathologist had used additional diagnostic techniques to assist with the diagnosis (such as immunohistochemistry to check if the glands were more likely benign or malignant).
   d. Detailed information provided about the diagnostic process would improve my acceptance of the report/diagnosis.
   e. Detailed information provided about the diagnostic process might cause me concern if I did not understand it.
   f. I am not interested in how the pathologist arrived at the diagnosis.

Strongly agree
Agree
Neutral
Disagree
Strongly disagree
Don’t know
Prefer not to say

4. In relation to the future potential for use of AI assistance in diagnosis, Please consider the following statements in relation to the **information that doctors (including pathologists) should record about AI assistance** in diagnosis and select one option:
   a. Doctors (including pathologists) should keep a record of all details of the AI-assistance output
   b. Doctors (including pathologists) should keep a record of human interpretation of the AI-assistance output only.
   c. Doctors (including pathologists) do not need to keep a record of any information about AI-assistance output
   d. Prefer not to say

Questions 5-10 aim to investigate awareness and attitudes to the potential for AI-assisted diagnosis generally, and in relation to prostate biopsy interpretation.

5. In relation to your **current level of experience** in the utility of AI for diagnosis in healthcare, please consider the following:

   a. I have an awareness of the use of AI in patient diagnosis in histopathology
   b. I have an awareness of the use of AI in patient diagnosis in other specialties such as radiology
   c. I have personal experience in using AI to assist diagnosis
   d. I am concerned about the use of AI for diagnosis in a clinical setting
   e. I look forward to the use of AI for diagnosis in a clinical setting

f. I would find it difficult to trust AI in making a diagnosis
g. I am concerned about the patient acceptance of AI for diagnosis
h. The use of AI for diagnosis is of no concern to me if the clinical team using it are comfortable with it

Strongly agree
Agree
Neutral
Disagree
Strongly disagree
Don't know
Prefer not to say

6. Please consider the following statements in relation to your degree of **comfort** in the potential use of clinically approved AI-assisted diagnosis for **prostate biopsy** reporting:

   a. I feel that it is the responsibility of the clinician (pathologist) to make the decision as to whether AI assistance is appropriate in the diagnosis of a prostate biopsy.
   b. I think that a patient would want to be able to decide whether AI assistance was used in the diagnosis of their biopsy.
   c. I would be comfortable with the idea of AI-assisted diagnosis in the interpretation of a prostate biopsy, but would want to know that a pathologist was responsible for the interpretation of the AI output and the final diagnostic report.
   d. I would be comfortable with the idea that AI-assisted interpretation of a prostate biopsy might replace a second opinion from another pathologist.
   e. I would feel comfortable with an AI diagnosis that a prostate biopsy was benign (no cancer), but would want a pathologist to double check this result was accurate.
   f. I would feel comfortable with an AI diagnosis that a prostate biopsy was benign (no cancer) without a pathologist checking that this result was accurate, if the pathologist was confident in the AI output.
   g. I would prefer that AI assistance is not used without explicit patient consent.
   h. I would prefer that all steps in prostate biopsy diagnosis were performed by a human without AI-assistance.
   i. If available, I would prefer that AI assistance is always used for diagnosing prostate cancer.
   j. I am concerned about the privacy of patient data if AI assistance is used.

Strongly agree
Agree
Neutral
Disagree
Strongly disagree
Don't know
Prefer not to say

7. Considering the **provision of information about AI-assisted diagnosis**, please indicate which of the following statements best applies to you:
    a. I would be comfortable with the use of AI-assisted diagnosis for a prostate biopsy if this was ‘approved’ for clinical use without my needing any additional information about it.
    b. I would be comfortable with the use of AI-assisted diagnosis for a prostate biopsy if this was ‘approved’ for clinical use, but would like to have access to information about the technology.
    c. I would not want AI to be used and I do not feel that any amount of additional information would influence my comfort level in relation to the use of AI in the diagnosis of a prostate biopsy.
    d. Prefer not to say

8. Please consider the following statements in relation to how well we understand **how AI-assistance works for diagnosis**:
    a. I am concerned that **patients** do not understand how AI-assistance works.
    b. I am concerned that **patient-facing doctors & wider clinical teams** (who are not pathologists) do not understand how AI-assistance works.
    c. I am concerned that **pathologists** do not understand how AI-assistance works.

Strongly agree
Agree
Neutral
Disagree
Strongly disagree
Don’t know
Prefer not to say

9. Please consider the following statements in relation to the **potential benefits** of AI assistance in prostate cancer diagnosis.
    a. I believe that AI assistance will reduce waiting times for prostate cancer diagnosis.
    b. I believe that AI assistance will improve the accuracy of prostate cancer diagnosis.
    c. I believe that use of AI assistance will increase **clinician** confidence in the prostate biopsy report / diagnosis
    d. I believe that use of AI assistance will increase **patient** confidence in the prostate biopsy report / diagnosis
    e. I believe that knowledge that AI assistance is used in the diagnosis of prostate biopsies may encourage patients to have a prostate biopsy
    f. I believe that AI assistance will replace human pathologists in the diagnosis of prostate cancer.
    g. I believe that AI assistance could improve efficiency of pathologists

Strongly agree
Agree

Neutral
Disagree
Strongly disagree
Don’t know
Prefer not to say

10. In relation to **what may impact on your acceptance** of the potential use of AI-assisted diagnosis for prostate biopsy reporting, please consider the importance of the following to you:

    a. Understanding how the technology works.
    b. Understanding how the technology was developed.
    c. Understanding who developed the technology (whether a pathologist has been involved).
    d. Understanding how the technology has been tested.
    e. Having access to data regarding performance of the technology (reliability compared with a pathologist).
    f. Understanding how a pathologist would use the technology in diagnosing a prostate biopsy.
    g. Understanding of who will be ultimately responsible for the diagnostic report if AI-assistance is used.

0 = Not at all important
1
2
3
4
5 = Very important
Don’t know
Prefer not to say

Any other comments (FREE TEXT)
